# Angiotensin‐converting enzyme open for business: structural insights into the subdomain dynamics

**DOI:** 10.1111/febs.15601

**Published:** 2020-11-02

**Authors:** Gyles E. Cozier, Lizelle Lubbe, Edward D. Sturrock, K. Ravi Acharya

**Affiliations:** ^1^ Department of Biology and Biochemistry University of Bath Bath UK; ^2^ Department of Integrative Biomedical Sciences Institute of Infectious Disease and Molecular Medicine University of Cape Town Cape Town South Africa

**Keywords:** angiotensin‐1‐converting enzyme, domain dynamics, enzyme mechanism, enzyme structure, metalloprotease, X‐ray crystallography

## Abstract

Angiotensin‐1‐converting enzyme (ACE) is a key enzyme in the renin–angiotensin–aldosterone and kinin systems where it cleaves angiotensin I and bradykinin peptides, respectively. However, ACE also participates in numerous other physiological functions, can hydrolyse many peptide substrates and has various exo‐ and endopeptidase activities. ACE achieves this complexity by containing two homologous catalytic domains (N‐ and C‐domains), which exhibit different substrate specificities. Here, we present the first open conformation structures of ACE N‐domain and a unique closed C‐domain structure (2.0 Å) where the C terminus of a symmetry‐related molecule is observed inserted into the active‐site cavity and binding to the zinc ion. The open native N‐domain structure (1.85 Å) enables comparison with ACE2, a homologue previously observed in open and closed states. An open S_2__S′‐mutant N‐domain structure (2.80 Å) includes mutated residues in the S_2_ and S′ subsites that effect ligand binding, but are distal to the binding site. Analysis of these structures provides important insights into how structural features of the ACE domains are able to accommodate the wide variety of substrates and allow different peptidase activities.

**Database:**

The atomic coordinates and structure factors for Open nACE, Open S2_S′‐nACE and Native G13‐cACE structures have been deposited with codes 6ZPQ, 6ZPT and 6ZPU, respectively, in the RCSB Protein Data Bank, www.pdb.org

## Graphical abstract

Angiotensin‐1‐converting enzyme (ACE) has broad substrate specificity and physiological functions. The first open structure of ACE N‐domain (nACE) presented here shows similar domain opening as ACE2 (close ACE homologue) that allows substrates to bind, and suggests extended binding site for endopeptidase activity. A second mutant nACE open structure examines the role of distal residues on ligand binding. In addition, a novel ACE C‐domain structure explains long peptide substrate binding.

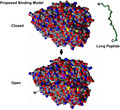

**Abbreviations**
ACE: angiotensin‐1‐converting enzyme
ACE2: angiotensin‐1‐converting enzyme 2
Ang I: angiotensin I
Ang II: angiotensin II
BPPb: bradykinin‐potentiating peptide b
cACE: angiotensin‐1‐converting enzyme C‐domain
nACE: angiotensin‐1‐converting enzyme N‐domain
RAAS: renin–angiotensin–aldosterone system
sACE: somatic angiotensin‐1‐converting enzyme
tACE: testis angiotensin‐1‐converting enzyme

## Introduction

Human angiotensin‐1‐converting enzyme (ACE, EC 3.4.15.1) occurs in two known isoforms, somatic (sACE) and testis (tACE), which contain two and one catalytically active ACE domains, respectively [1, 2, 3]. The two domains in sACE are referred to as the N‐ and C‐domains (nACE and cACE, respectively). ACE has been classified as a zinc dipeptidyl carboxypeptidase based on the most well‐known functions of sACE in cardiovascular physiology [4]. As part of the renin–angiotensin–aldosterone system (RAAS), it converts angiotensin I (Ang I) to the vasoactive peptide hormone angiotensin II (Ang II) [5] and decreases vasodilation by cleaving the peptide bradykinin (BK) [6].

The role of sACE has subsequently been expanded with the discovery of a wider range of substrates, including the ability to act as a tripeptidyl carboxypeptidase, endopeptidase and N‐terminal peptidase, and a potential role as a signalling molecule [7]. sACE has reported roles in fibrosis [8, 9], myelopoiesis [10], erythropoiesis [11], haematopoiesis [12], immunity [13, 14] and renal development and function [15]. In addition to Ang I, peptides that have been identified as substrates for these and potentially other roles include N‐acetyl‐Ser‐Asp‐Lys‐Pro (AcSDKP), substance P, enkephalins, luteinising hormone‐releasing hormone, kinins, the amyloid‐beta peptide (Aβ), neurotensin and formyl‐Met‐Leu‐Phe [16, 17, 18, 19, 20, 21]. A recent study which used mass spectrometry to study ACE activity in mouse plasma identified more than 240 potential substrates or products of ACE, demonstrating the vast promiscuity of this enzyme [22]. Furthermore, ACE has been implicated in the immune response through cleavage of Ang I as well as an unknown substrate [23].

**Fig. 1:**
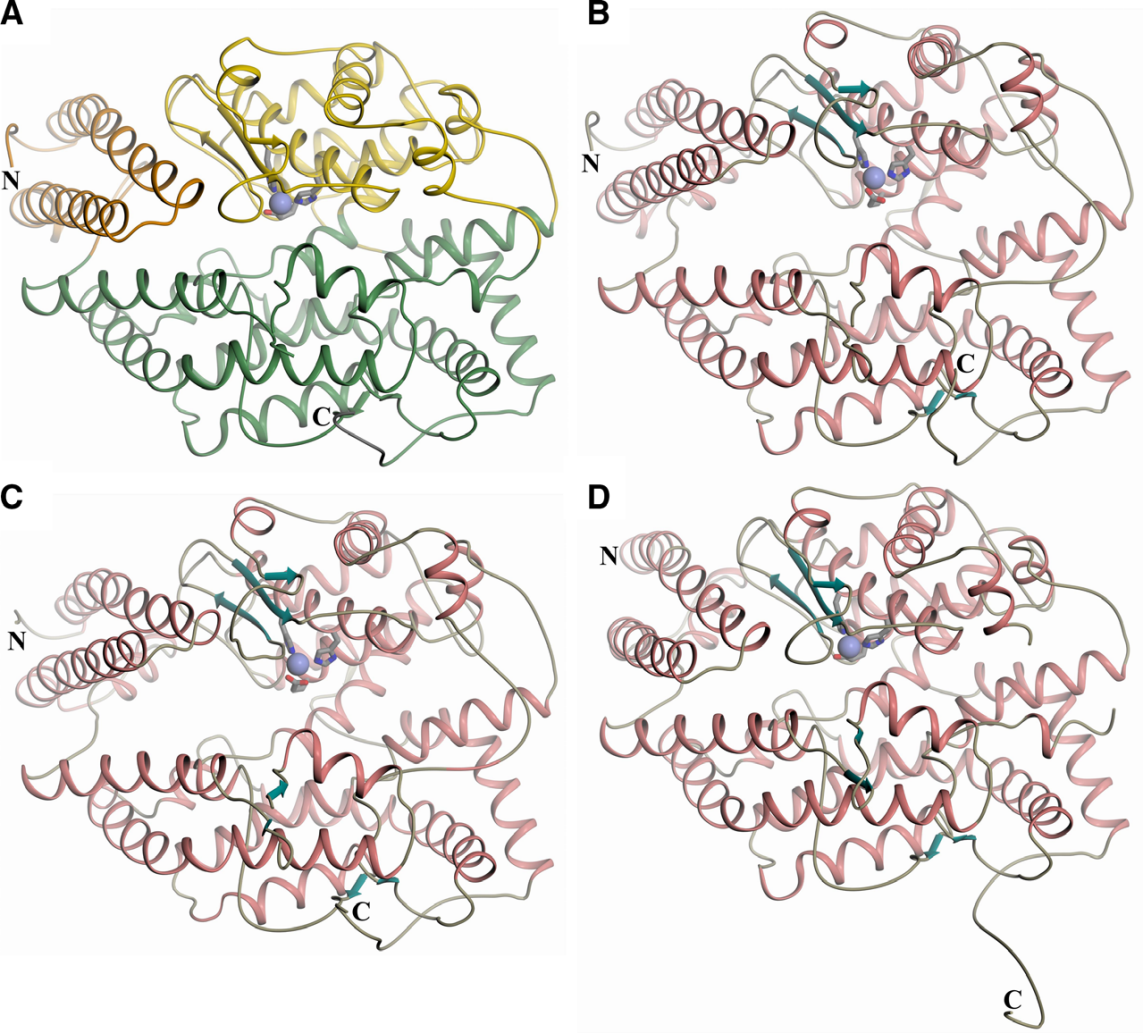
Schematic structure representations. Crystal structures of (A) typical closed nACE (PDB code‐6F9V [32]). (B) open nACE. (C) Open S_2__S′‐nACE and (D) native cACE. The active‐site zinc ion is depicted as a lilac sphere, zinc‐binding residues (His‐, His‐ and Glu‐) as sticks, with α‐helices and β‐strands (in panels B–D) coloured rose and dark cyan, respectively. Panel A shows subdomains 1 and 2 in orange (the lid‐like region in dark orange) and green colour, respectively. Loop regions have been smoothed for clarity. Figure was generated using CCP4mg [47].

The promiscuity of sACE's function can partially be explained by the two ACE domains which share 89% identical active‐site region sequences (60% overall sequence similarity), yet exhibit different substrate specificities. Indeed, structurally nACE and cACE have similar α‐helical ellipsoid shapes containing a two‐lobed binding site cavity with an active‐site zinc ion bound by two histidines and one glutamate residue located at the junction of the two lobes [24, 25] (Fig. 1A). The ellipsoid can be divided into two subdomains with subdomain 1 containing a ‘lid‐like’ region formed by the N‐terminal 100 residues. However, it is still unclear how sACE can achieve such variation in exo‐ and endopeptidase activity. To date, all structures of nACE and cACE, whether they are native or in complex with a peptide or inhibitor, have adopted a ‘closed conformation’. In contrast, ACE2 (a single‐domain enzyme), which is a close homologue of ACE, has been crystallised in both ‘closed’ and ‘open’ conformations, which explain how substrates and products can enter and exit the active site [26]. The two subdomains of ACE2 open in a ‘clam shell’‐like manner, resulting in a deep groove leading to the binding site. There have been several molecular dynamics studies that have indicated that ACE is able to open in a similar way [27, 28, 29]. In particular, the lid was identified as one of the six hinge regions within each domain using normal mode analysis [29]. However, the mechanism of how ligands enter and exit the binding site remains unclear.

Here, we present the first ‘open’ structures of an sACE domain—both native nACE and the S_2__S′‐mutant enzyme. This nACE mutant contains eight residues mutated to their cACE equivalents (Y369F and R381E from the S_2_ subsite, T358V from the S_1_′ subsite, and S260T, E262S, D354E, S357V and E431D located in the S′ lobe distal from the S_2_′ subsite), which has previously been used to show that residues distal from the binding site effect ligand affinity without significantly affecting nACE structure [27, 30]. In addition, we have crystallised a cACE structure where the C terminus of a symmetry‐related molecule is inserted into the binding cavity, where it interacts with the active‐site zinc ion. Analysis of these high‐resolution structures not only shows precisely how the ACE domains of sACE structurally open, but also provides insights into how these domains achieve catalysis of such a wide range of substrates with exo‐ and endopeptidase activity, and gives further information on how residues at the subdomain interface can contribute to domain opening and closing.

## Results

### Open structure of nACE

**Fig. 2:**
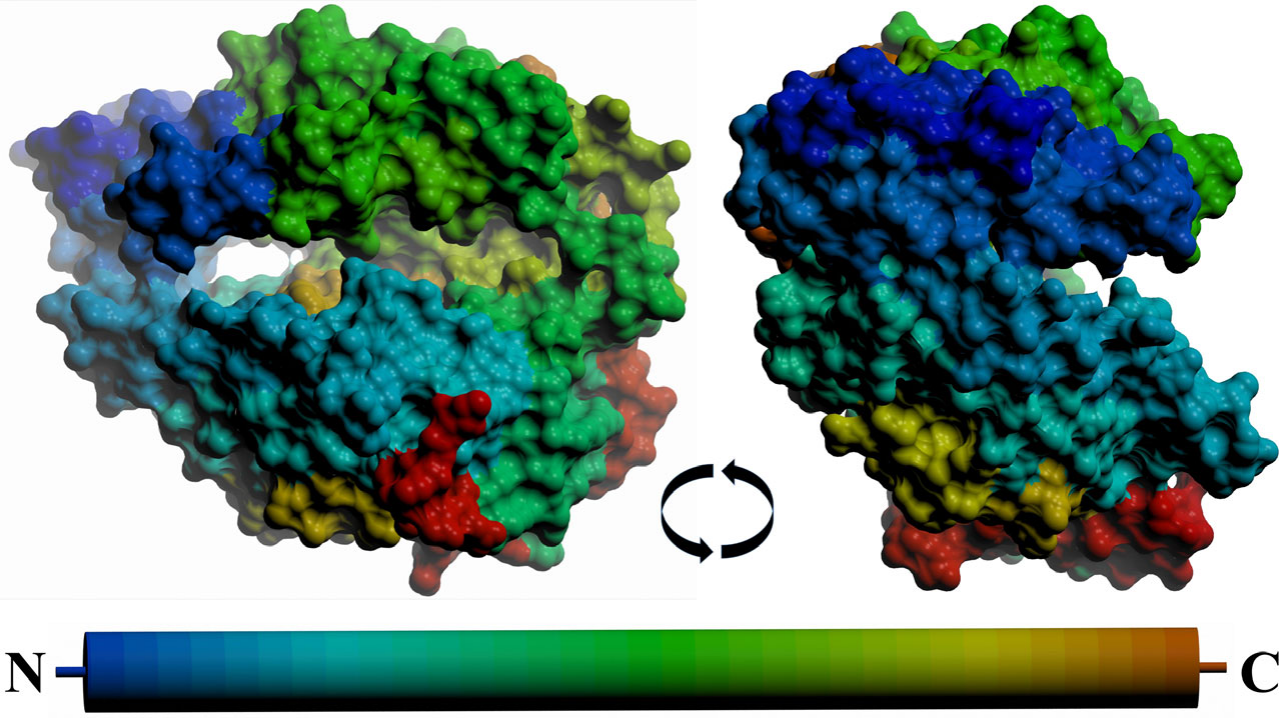
Surface representation of the open nACE structure. Two views are shown rotated 90° about the *x*‐axis, with the peptide chain coloured from blue (N terminus) to red (C terminus) as indicated by the cylinder. This shows that the two subdomains have opened to allow access to the binding site cavity. Figure was generated using CCP4mg [47].

Native nACE is heavily glycosylated, with 9 of the 10 predicted N‐linked sites modified. In the clone used for crystallisation (N389‐nACE [31]), six of these are mutated to glutamine residues leaving only Asn‐45, Asn‐416 and Asn‐480 glycosylated resulting in an active enzyme that readily crystallises. During a typical cocrystallisation with inhibitor, which usually produces chunky, rod‐like crystals, a different crystal with plate morphology was observed growing near the edge of the hanging drop. Although this morphology had not been seen previously, it could be repeated by seeding of 1‐day‐old drops from crystals that grew after the original crystal had been picked for X‐ray diffraction data collection. The usual rod‐like crystals have a *P*1 space group, with either two or four molecules in the asymmetric unit and cell dimensions (bond length and angles) of 73, 77, 88 Å and 89, 64, 75°, and 73, 102, 115 Å and 85, 85, 81°, respectively. The new plate‐shaped crystal also grew in the *P*1 space group with four molecules in the asymmetric unit, but with cell dimensions of 75, 100, 129 Å and 98, 90, 111° (Table 1). Molecular replacement using the usual search model from nACE in complex with sampatrilat (PDB code‐6F9V) [32] gave a partial result, but it was clear that only about half the structure fitted the electron density. Interestingly, while one subdomain fitted the density well, the other did not, and it was clear from the density that this was because the structure was in a more open conformation than has previously been observed. The molecular replacement was repeated, but with individual subdomains given as search models, which produced an initial model that fitted the electron density well. After refinement, the final model (resolution of 1.85 Å, *R*_work_ of 0.184 and *R*_free_ of 0.214) showed typical nACE secondary structure elements being mostly α‐helical and arranged in two subdomains, but these subdomains were open in a ‘clam shell‐like’ conformation in all four molecules of the asymmetric unit (Figs 1B and 2).

**Table 1:** X‐ray data collection and refinement statistics. Inner shell, overall and outer shell statistics are given in square brackets, unbracketed and round brackets, respectively.

	Open nACE	Open S_2__S′‐nACE	Native G13‐cACE
Resolution (Å)	[92.26–10.13] (1.88–1.85)	[91.40–14.55] (2.85–2.80)	[84.90–8.94] (2.05–2.00)
Space group	P1	P1	P212121
Cell dimensions (*a*, *b*, *c*) angles (α, β, γ)	74.7, 99.9, 128.7 Å 97.8, 90.2, 111.1°	73.7, 99.3, 128.0 Å 98.6, 89.6, 111.2°	58.5, 84.9, 128.2 Å 90.0, 90.0, 90.0°
Molecules/asymmetric unit	4	4	1
Total/unique reflections	1 987 984 286 723	567 969 81 448	565 562 43 959
Completeness (%)	[99.7] 97.7 (96.3)	[99.7] 99.0 (98.1)	[99.9] 100.0 (100.0)
*R*_merge_	[0.044] 0.134 (2.474)	[0.060] 0.264 (1.837)	[0.062] 0.301 (2.959)
*R*_pim_	[0.018] 0.055 (1.010)	[0.025] 0.107 (0.774)	[0.019] 0.087 (0.845)
<*I*/σ(*I*)>	[28.8] 7.7 (0.8)	[15.1] 4.9 (0.8)	[24.6] 6.3 (0.9)
CC_1/2_	[0.998] 0.996 (0.546)	[0.998] 0.985 (0.617)	[0.997] 0.996 (0.485)
Multiplicity	[6.8] 6.9 (6.9)	[6.6] 7.0 (6.6)	[11.6] 12.9 (13.3)
Refinement statistics			
*R*_work/_ *R*_free_	0.184/0.214	0.223/0.278	0.191/0.235
RMSD in bond lengths (Å)	0.009	0.002	0.003
RMSD in bond angles (°)	0.773	0.526	0.642
Ramachandran statistics (%)			
Favoured	97.92	96.30	98.11
Allowed	2.04	3.66	1.89
Outliers	0.04	0.04	0.00
Average *B*‐factors (Å^2^)			
Protein	38.9	64.5	35.3
Ligand	58.4	73.8	57.6
Water	41.5	46.6	36.2
Number of atoms			
Protein	20 051	19 624	4812
Ligand	520	262	157
Water	1672	55	279
PDB code	6ZPQ	6ZPT	6ZPU

**Fig. 3:**
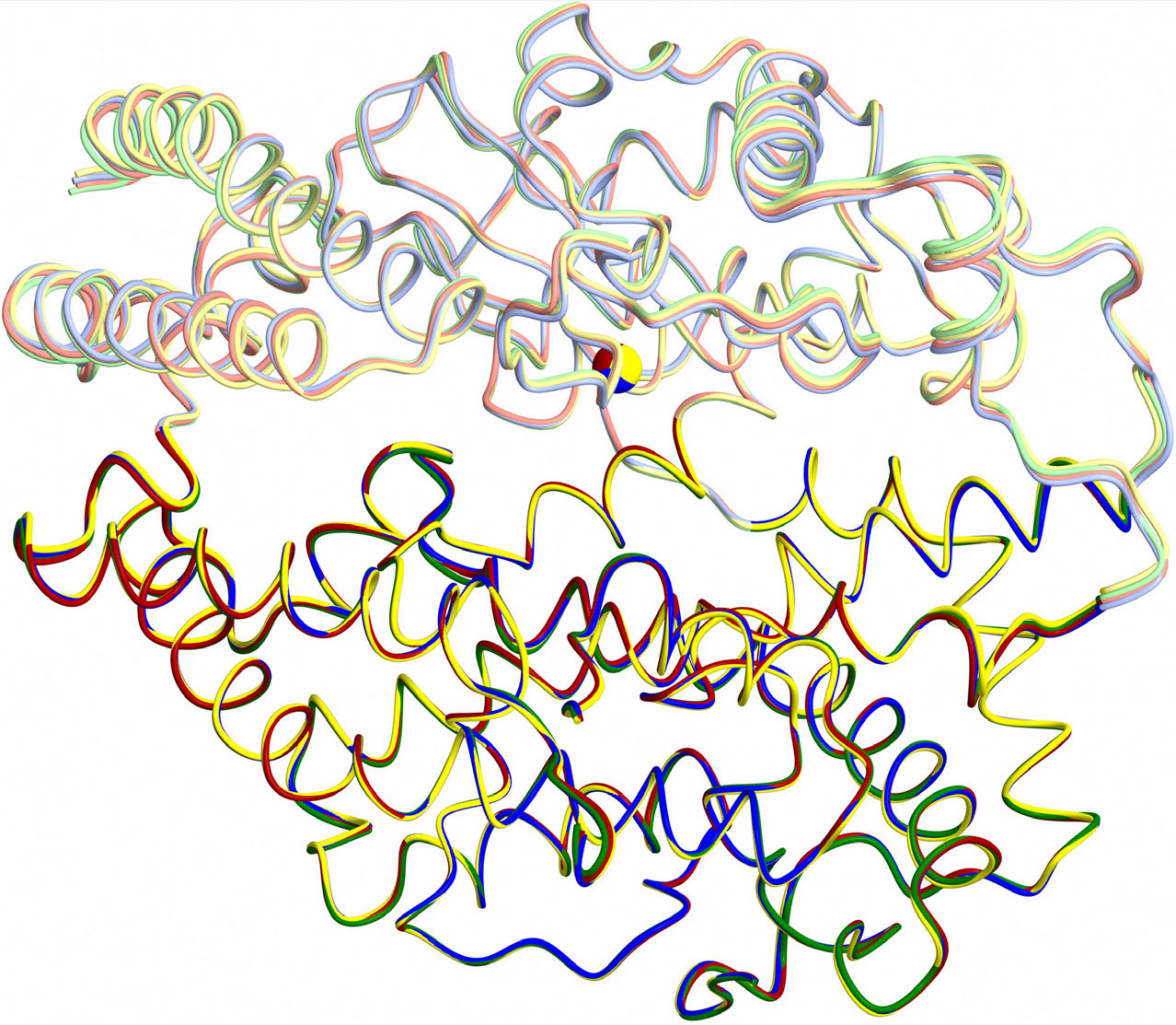
Variation in opening of molecules on nACE in the asymmetric unit. The four molecules in the open nACE structure asymmetric unit are overlaid using the subdomain 2 residues. This highlights differences in the subdomain 1 overlay indicating a small variation in degree of opening. Subdomains 1 and 2 are shown in lighter and darker colours, respectively. Figure was generated using CCP4mg [47].

An overlay of the four molecules in the asymmetric unit of the open nACE structure showed that in addition to the flexibility of the lid‐like region that is usually observed in ACE domain structures, there is also a small variation in the degree of openness (Fig. 3). However, this only results in a maximum shift of about 1 Å at the most flexible regions at the lip of subdomain 1, and the RMSD values for 605 Cα atoms observed in all structures range from 0.14 to 0.35 Å.

**Fig. 4:**
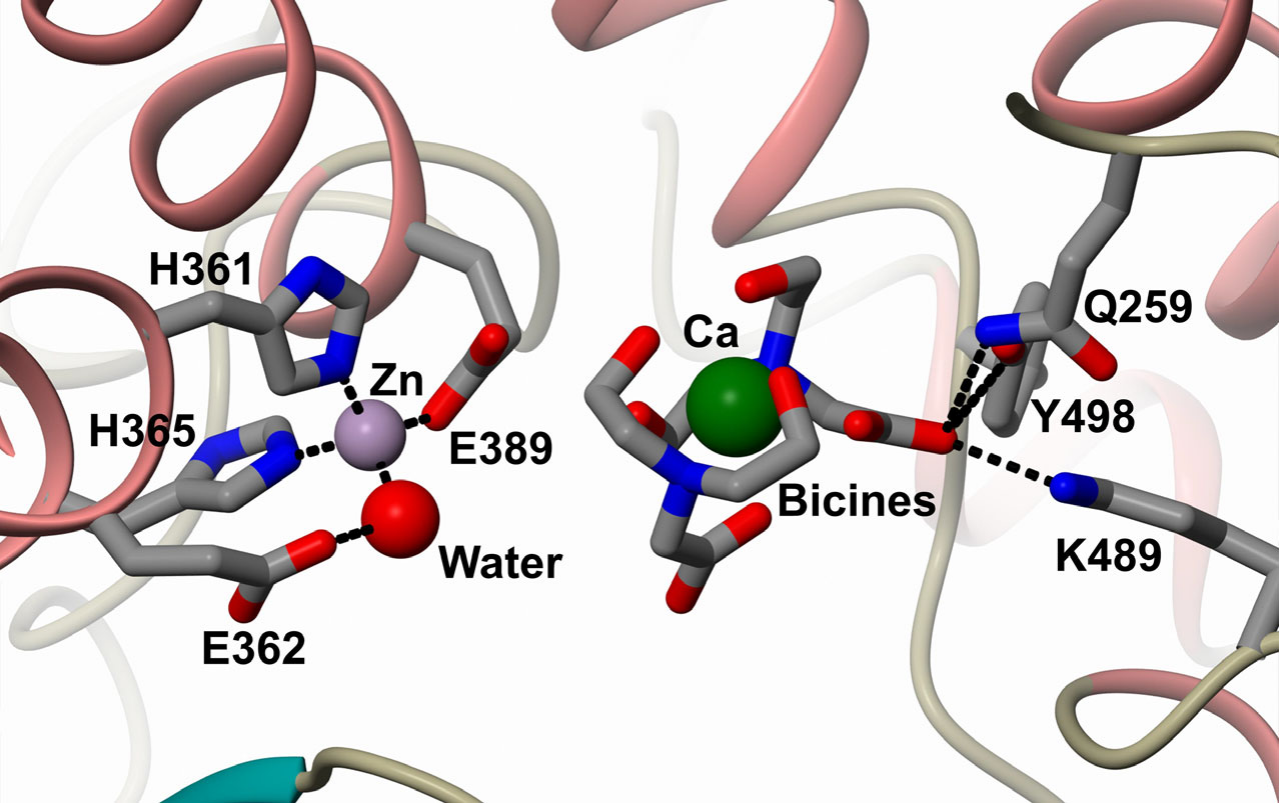
Open nACE structure active site. Close‐up view of the zinc and substrate C terminus binding sites of the nACE open structure. The zinc ion interacts with the conserved two histidine and one glutamate residues, with the coordination sphere completed by a water molecule that also binds to the catalytic Glu‐362. The substrate C terminus binding site is occupied by a calcium ion coordinated by two bicine molecules. α‐helices and β‐strands coloured rose and dark cyan, respectively. Figure was generated using CCP4mg [47].

The active‐site zinc ion was coordinated in the usual manner for ACE domains to His‐361, His‐365 and Glu‐389, with its coordination sphere completed by a water molecule (Fig. 4). This water molecule also interacts with the proposed catalytic Glu‐362 residue, and therefore, this structure is likely showing nACE with the catalytic water in the resting state.

Residues Gln‐259, Lys‐489 and Tyr‐498 form the binding pocket for the carboxy terminus of nACE peptide substrates, and even in structures without added ligand, this pocket always binds something from expression, purification or crystallisation reagents. In the present open structure, a bicine molecule from the crystallisation buffer is bound, with the carboxy group being a peptide C terminus mimic (Fig. 4). The bicine molecules also form half of a coordination sphere for a metal ion, with a calcium ion from the crystallisation buffer fitting optimally the electron density. The electron density for the other half of the calcium's coordination sphere shows variation between the different molecules in the asymmetric unit, where in one molecule a second bicine molecule fits well (Fig. 4), whereas in the other molecules water molecules and a PEG were modelled. This reflects that there is probably mixed occupancy of molecules completing the calcium coordination.

Typically, in nACE structures the observed glycosylation electron density on Asn‐45 and Asn‐480 is limited to between 1 and 3 carbohydrates, whereas on Asn‐416 carbohydrates up to and beyond the branching point of the β‐D‐mannose can be modelled. In this open structure, while between 1 and 3 carbohydrates can still be modelled for the Asn‐45 and Asn‐480 glycosylation, the electron density observed on Asn‐416 is very weak such that no carbohydrate molecule could be confidently modelled on any of the chains in the asymmetric unit.

A single structurally important chloride ion is always observed bound to Tyr‐202, Arg‐500 and a water molecule in the closed nACE structures. This has been linked to the chloride‐dependent activity of nACE with certain substrates, possibly due to the interaction via a water molecule between Arg‐500 and the zinc‐binding Glu‐389 residue. In the open nACE structure presented in the present study, the chloride ion was observed in all molecules of the asymmetric unit and is bound in an identical manner. However, due to the subdomain movement, Arg‐500 no longer interacts with Glu‐389 via a single water molecule, although there is a network of three water molecules linking them. Therefore, this closer interaction between Arg‐500 and Glu‐389, and any subsequent chloride‐dependent activity, requires the enzyme to return to the closed conformation after a substrate has entered the active site.

### Structure of S_2__S′‐active‐site mutant nACE

Previously, it had been suggested that some of the mutated residues of the S_2__S′‐mutant nACE (containing cACE equivalent mutations Y369F and R381E from the S_2_ subsite, T358V from the S_1_′ subsite, and S260T, E262S, D354E, S357V and E431D located in the S′ lobe distal from the S_2_′ subsite) could be involved in the hinge movement of nACE [27, 30]. Crystallisation trials of N389‐S_2__S′‐mutant nACE (the same minimal glycosylation as for native nACE) using the typical conditions used for nACE failed to produce any crystals without added ligand, even after seeding from native nACE crystals. Producing apo crystals, and potentially in the open conformation, would allow analysis of these mutated residues in order to further examine their role in subdomain dynamics without the interference of bound ligand. Therefore, seeding experiments of the S_2__S′‐mutant were performed using the plate crystals of open conformation nACE. This resulted in numerous plate crystals of the S_2__S′‐mutant, which like the native nACE open structure were also in the *P*1 space group with similar cell dimensions (lengths of 74, 99 and 128 Å, and angles of 99, 90 and 111°), and there were four molecules in the asymmetric unit (Table 1). The open structure of nACE was used as the molecular replacement model, resulting in a final structure at 2.80 Å resolution with *R*_work_ and *R*_free_ of 0.223 and 0.278, respectively. Like nACE, the overall structure was an open ‘clam shell‐like’ arrangement of two subdomains largely consisting of α‐helical secondary structure (Fig. 1C). There is minimal variation between the four molecules in the asymmetric unit with RMSD values for 591 Cα atoms observed in all structures ranging from 0.19 to 0.40 Å.

The active‐site zinc ion was observed with the typical coordination to His‐361, His‐365 and Glu‐389, with the probable catalytic water molecule coordinated to the zinc ion and the catalytic Glu‐362. Likewise, the chloride ion was observed having identical interactions with Tyr‐202 and Arg‐500 as observed with nACE, although no water molecule could be modelled likely due to the lower resolution.

A bicine molecule was identified in the prime lobe of the binding site, similar to that observed in nACE, with the carboxy group of bicine interacting with Gln‐259, Lys‐489 and Tyr‐498. A calcium ion was modelled coordinating to the bicine, but due to the lower resolution the complete calcium coordination sphere was not observed, resulting in only some water molecules being included.

The glycosylation on Asn‐45 and Asn‐480 extended to up to three carbohydrates, although only one or two could be modelled. However, as with the open structure of nACE, the electron density around Asn‐416 was very weak, and even the amino acid side chain was poorly defined; therefore, no glycosylation could be modelled.

### Structure of novel native cACE

Native cACE is heavily glycosylated with six of the seven predicted N‐linked sites bound to glycan chains, and similar to nACE, an active minimally glycosylated mutant (g13‐cACE glycosylation on Asn‐72 and Asn109) is used to aid crystallisation [29]. Like previous cACE structures [29, 32, 33], the native g13‐cACE crystallised in the *P*2_1_2_1_2_1_ space group with one molecule in the asymmetric unit and the resulting structure was determined at 2.0 Å resolution (Table 1). This structure showed the typical closed conformation of a mostly α‐helical ellipsoid, with a buried two‐lobed cavity in the centre (Fig. 1D). The active site is located at the junction of these lobes with the catalytic zinc ion flanked by the S′ and S subsites. As observed previously, the usual two chloride ions are bound to g13‐cACE. These are required for ACE activity and have an effect on inhibitor affinity. The first of these binding sites is formed by residues Arg‐186, Trp‐485 and Arg‐480 and a water molecule, whereas the second contains interactions with Tyr‐224, Arg‐522 and a water molecule.

Minimally glycosylated g13‐cACE has been used for structural studies because it retains its activity and high reproducibility in crystal growth for structural studies. Only the first (Asn‐72) and third (Asn‐109) of the six glycosylation sites are retained, and both of these sites show clear density for carbohydrates, with three and eight sugars modelled, respectively. The C terminus of peptide substrates and inhibitor carboxy/amide groups bind in the S_2_′ subsite which is comprised of residues Gln‐281, Lys‐511 and Tyr‐520. In the native structure presented here, an acetate molecule from the crystallisation conditions is observed in the S_2_′ subsite.

**Fig. 5:**
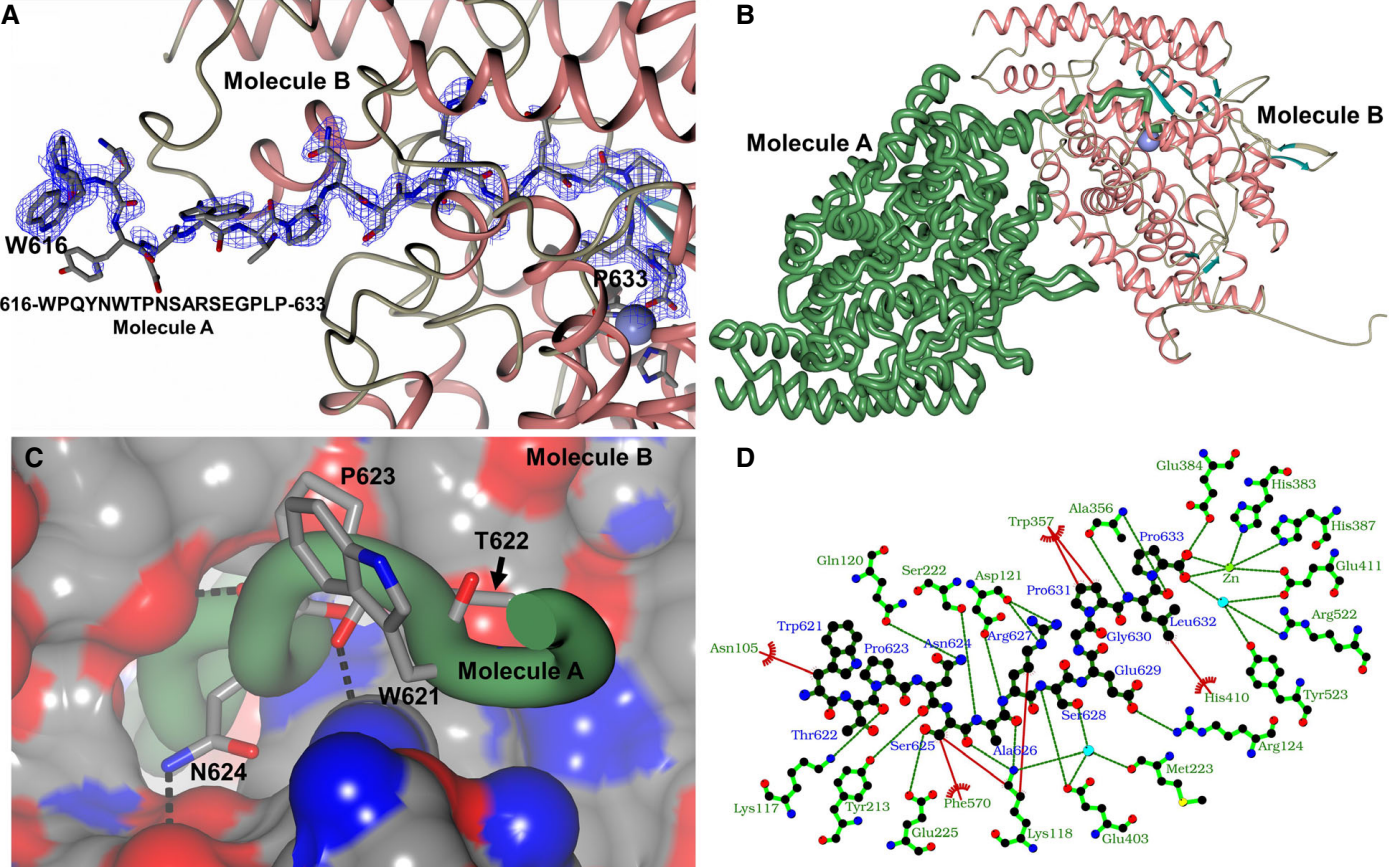
Novel cACE structure with C terminus insertion. A native G13‐cACE crystal structure shows the insertion of a symmetry‐related molecule's C terminus into the nonprime lobe and interaction with the active‐site zinc ion. Zinc ions are shown as lilac spheres, with α‐helices and β‐strands shown in rose and dark cyan, respectively. (A) Close‐up view of the C terminus insertion overlaid with the final 2mFo –DFc (blue, contoured at 1σ level) electron density map. (B) Arrangement of G13‐cACE crystal packing showing molecule A (green worm) with its C terminus orientated to insert into the nonprime lobe of symmetry‐related molecule B. (C) Close‐up view of C terminus insertion showing interacting residues of molecule A (green worm—residues **619**–**633** were used to generate figure, but not all residues are visible within the cavity) at the surface of symmetry‐related molecule B. (D) Native G13‐cACE LigPlot representation showing the binding of the C terminus of molecule A (dark green) within the nonprime binding lobe of symmetry‐related molecule B (light green). H‐bond/electrostatic interactions are shown in green, hydrophobic interactions in red and water molecules as red spheres. Residues solely involved in hydrophobic interactions are represented by red, semicircular symbols. Panels A, B and C were generated using CCP4mg [47] and Panel D with LigPlot+ [49].

Angiotensin‐1‐converting enzyme substrates and inhibitors bound in the active site have interactions with the catalytic zinc ion, and in native structures, this zinc completes its coordination sphere with molecules from the purification/crystallisation media or water molecules. Examination of the electron density adjacent to the zinc ion in the native structure presented here showed density for a substantial ligand extending into the nonprime binding lobe (Fig. 5A). During refinement, it became clear that this was the C terminus tail of a symmetry‐related molecule that had inserted through a small opening into the nonprime lobe that is adjacent to the ‘lid‐like’ region (Fig. 5B). While this is likely to be a crystallisation artefact, it does have implications for substrate binding and potentially product release, which are discussed in detail below.

Residues **Trp‐621**, **Thr‐622** and **Pro‐623** (for clarity in the following text, the C‐terminal residues of molecule A are shown in bold, and these insert into the nonprime lobe of the adjacent symmetry‐related molecule B whose residues are shown in italics) form crystal packing interactions on the surface of the adjacent symmetry‐related molecule B ellipsoid at the entrance to the small hole leading into the nonprime binding lobe (Fig. 5C). At residue **Asn‐624,** the C terminus begins to insert into the hole with residues **Ser‐625** and **Ala‐626** completing the insertion through a small channel and the remaining C‐terminal residues (**Arg‐627** to **Pro‐633**) are located inside the cavity. Most of the C‐terminal residues show interactions with the symmetry‐related molecule B apart from **Pro‐623** and **Gly‐630** which do not interact (Fig. 5D).

In detail, **Trp‐621** and **Thr‐622** at the channel entrance have a hydrophobic interaction with *Asn‐105* and a hydrogen bond with *Lys‐117*, respectively. Passing through the channel, **Asn‐624** has hydrogen bonds with *Gln‐120* and *Tyr‐213*. **Ser‐625** has hydrogen bonds from its side chain and backbone oxygen atoms with *Glu‐225* and *Lys‐118*, respectively, as well as hydrophobic interactions with *Lys‐118* and *Phe‐570*. Completing the passage through the channel, **Ala‐626** has two hydrogen bonds from its backbone oxygen and nitrogen atoms with residues *Lys‐118* and *Ser‐222*, respectively. **Arg‐627** lies just inside the cavity where its guanidine group interacts with the backbone oxygen of *Asp‐121*, its side chain has a hydrophobic interaction with *Lys‐118*, and its backbone nitrogen interacts with the side chain of *Asp‐121*. The electron density and B‐factors show that residues **Ala‐626** to **Gly‐630** are more flexible than residues **Pro‐631** to **Pro‐633**, and this is reflected in the number of interactions observed. While both oxygens of the carboxylic group of *Glu‐430* are within 3.4 Å of the **Ser‐628** backbone nitrogen, only one is in the ideal orientation for a strong interaction. In addition, the other interactions are water‐mediated hydrogen bonding between the side chain of **Ser‐628** with *Lys‐118*, *Met‐223* and *Glu‐403*. **Glu‐629** has a solitary hydrogen bond with *Arg‐124*, while **Gly‐630** has no interactions within the cavity. In contrast, **Pro‐631** has extensive hydrophobic interactions with *Trp‐357*, and the side chain of **Leu‐632** has a hydrophobic interaction with *His‐410* and two hydrogen bonds between its backbone and the backbone of *Ala‐236*. Finally, **Pro‐633** has extensive interactions including a bidentate interaction with the zinc ion, another hydrogen bond with the catalytic *Glu‐384* and interactions via a water molecule with *Glu‐411*, *Arg‐522* and *Tyr‐523*.

## Discussion

### Analysis of subdomain dynamics for nACE opening

The majority of nACE, cACE and ACE homologue (AnCE, a homologue from *Drosophila melanogaster,* and AnoACE2, a homologue from *Anopheles gambiae*) structures determined to date have been solved in complex with substrates or inhibitors bound in the active site. This has favoured the closed conformation of ACE due to the number of interactions between the ligand and residues from both subdomains. This is particularly apparent with the zinc‐binding residues located in subdomain 1 and the substrate carboxy terminus binding site in subdomain 2, where the binding of substrate or inhibitor would help pull the two subdomains together into the closed conformation. The open structure presented here for nACE has only a water molecule bound to the zinc ion and a calcium/bicine complex bound to the substrate carboxy terminus binding site. While this fills the space between the two subdomains, there are no strong interactions between the two subdomains in the active‐site region, which may favour the open conformation observed. A comparison of the open and closed nACE structures provides information on how substrates and inhibitors can enter the active site, and details of the subdomain dynamics that are required during the domain opening.

**Fig. 6:**
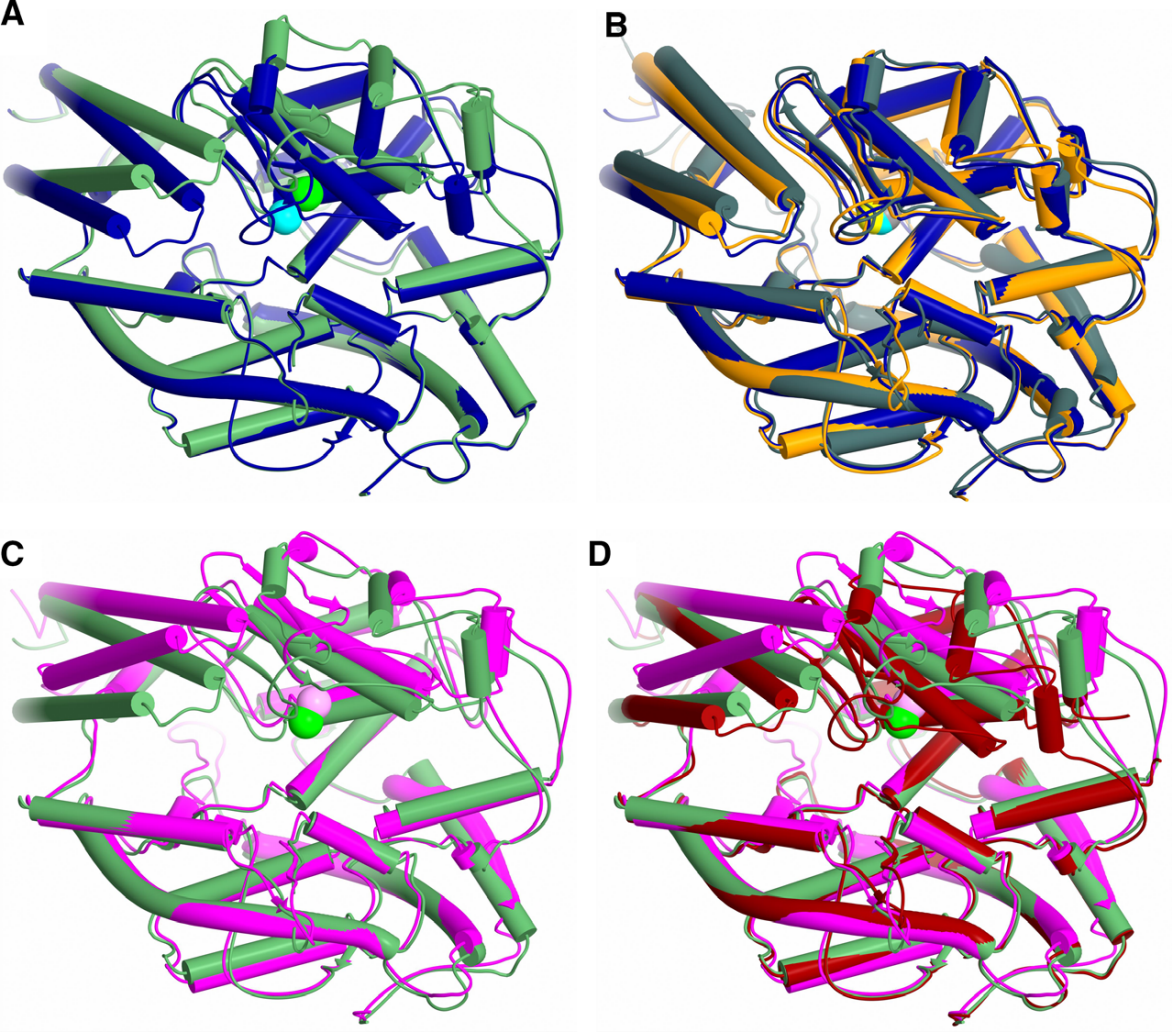
Schematic representation showing structural overlays. Structural overlays of (A) closed nACE (blue) and open (green) nACE, (B) closed nACE (blue), closed cACE (orange) and closed ACE2 (grey), (C) open nACE (green) and open ACE2 (magenta) and (D) open nACE (green), open cACE (dark red) and open ACE2 (magenta). Zinc atoms are shown as spheres and coloured lighter than their corresponding protein. α‐helices and β‐strands are depicted as cylinders and arrows, respectively, with loop regions shortened for clarity. PDB codes for closed nACE (sampatrilat complex), closed cACE (omapatrilat complex), closed ACE2 (MLN‐4760 complex), open cACE (BPPb complex) and open ACE2 (native) are 6F9V [32], 6H5W [37], 1R4L [26], 4APJ [34] and 1R42 [26], respectively. Figure was generated using CCP4mg [47].

**Fig. 7:**
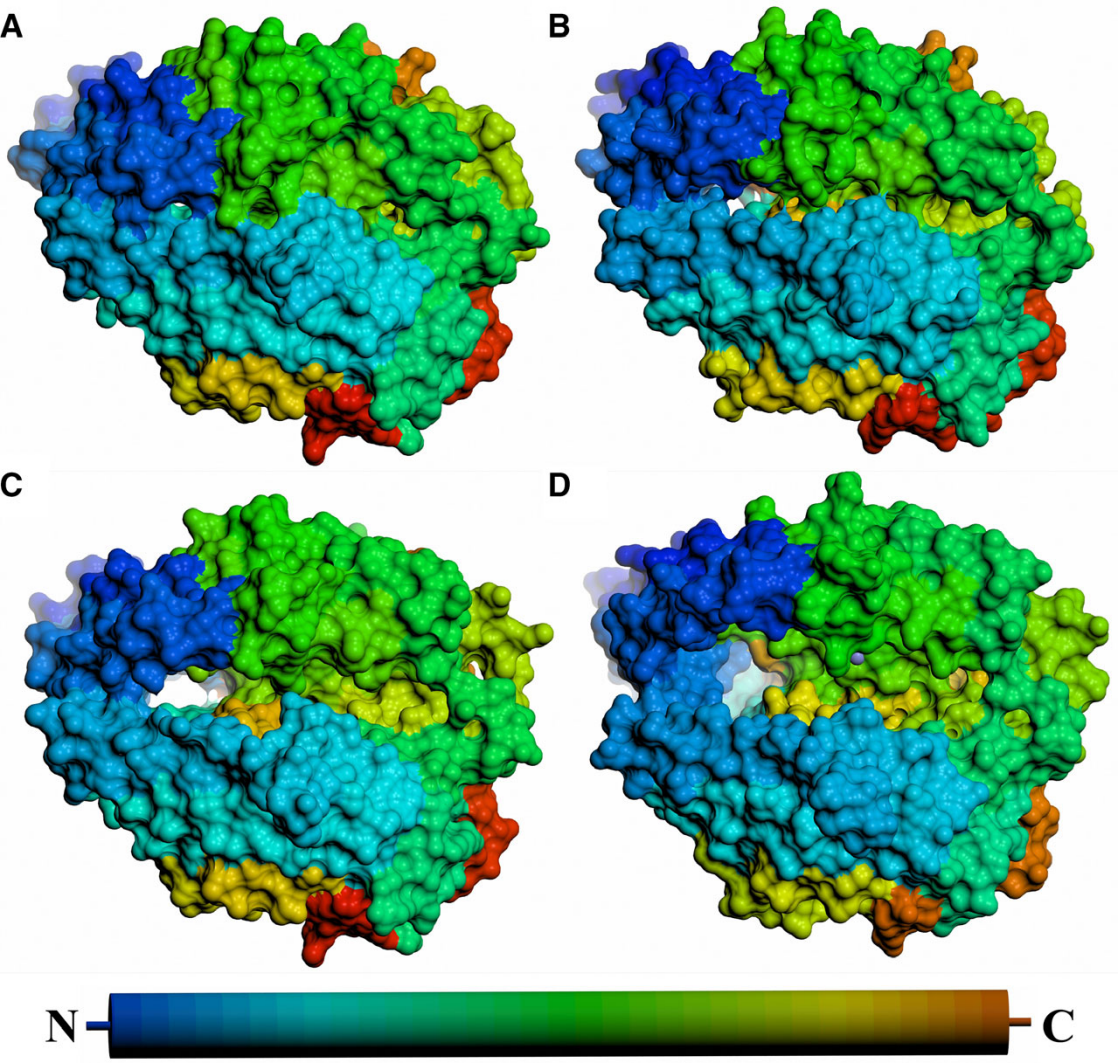
Surface representation comparison of ACE/ACE2 opening. Surface representations of (A) closed nACE, (B) BPPb‐cACE, (C) open nACE and (D) ACE2 structures. The peptide chains are coloured from blue (N terminus) to red (C terminus) as indicated by the cylinder, and the active‐site zinc (if visible) is a lilac sphere. PDB codes for closed nACE (sampatrilat complex), open cACE (BPPb complex) and open ACE2 (native) are 6F9V [32], 4APJ [34] and 1R42 [26], respectively. Figure was generated using CCP4mg [47].

An overlay of the open and closed nACE structures (Fig. 6A) shows that the two subdomains open in a ‘clam shell‐like’ way, resulting in a deep groove leading to the active site. This, and subsequent overlays, was generated by superposing subdomain 2 of the structures (nACE residues 98–271, 418–506 and 567–601) to enable visualisation of the relative movement of subdomain 1 (nACE residues 11–97, 272–417 and 507–566) as the ACE domain opens. Based on the overlays, the RMSD values for the Cα atoms that are observed in each pair of structures are shown in Table 2. The resulting open and closed nACE structures had an RMSD of 2.39 Å for 587 Cα atoms of the two subdomains that are observed in both structures. According to surface depiction of the closed conformation, there is almost no view into the binding cavity (Fig. 7A), whereas in the open structure (Fig. 7C), there is access across the full length of the nACE domain into both the prime and nonprime lobes. There are some residue side chains that appear to partially block this new opening in the crystal structure, but they are likely to be flexible and therefore could move to allow ligand entry. This would enable peptide substrates or inhibitors to access the binding site in an elongated linear conformation, after which the subdomains could close to envelop the ligand and adopt the catalytic closed structure.

**Table 2:** Comparison of open and closed ACE/ACE2 structures. RMSD values (Å) for Cα atoms observed in both structures of each comparison shown in brackets. Values were generated using the ‘Structure Alignment and Superposition with Gesamt’ program on CCP4 cloud.

	cACE Closed	ACE2 Closed	nACE Open	cACE Open	ACE2 Open
nACE Closed	1.163 (585)	2.416 (570)	2.390 (587)	1.903 (562)	4.622 (570)
cACE Closed	–	1.921 (578)	2.393 (573)	0.979 (580)	4.187 (578)
ACE2 Closed	–	–	2.991 (571)	2.618 (566)	3.631 (592)
nACE Open	–	–	–	2.250 (563)	2.875 (571)
cACE Open	–	–	–	–	4.163 (566)

	nACE Closed	S_2__S′‐nACE Open	nACE Open
S_2__S′‐nACE Closed	0.663 (586)	2.341 (586)	2.341 (584)
nACE Closed	–	2.314 (585)	2.390 (587)
S_2__S′‐nACE Open	–	–	0.425 (586)

**Fig. 8:**
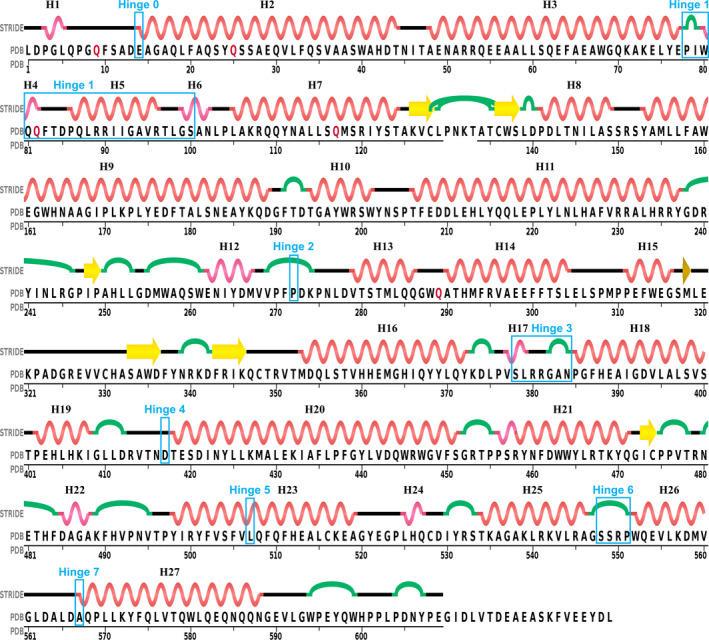
N389‐nACE sequence.Sequence and secondary structure (generated using STRIDE [50] from sampatrilat complex PDB 6F9V [32]) of nACE. Helices (H) are numbered sequentially, β‐strands and turns are indicated in yellow and green, respectively, and hinges are shown in blue boxes.

**Fig. 9:**
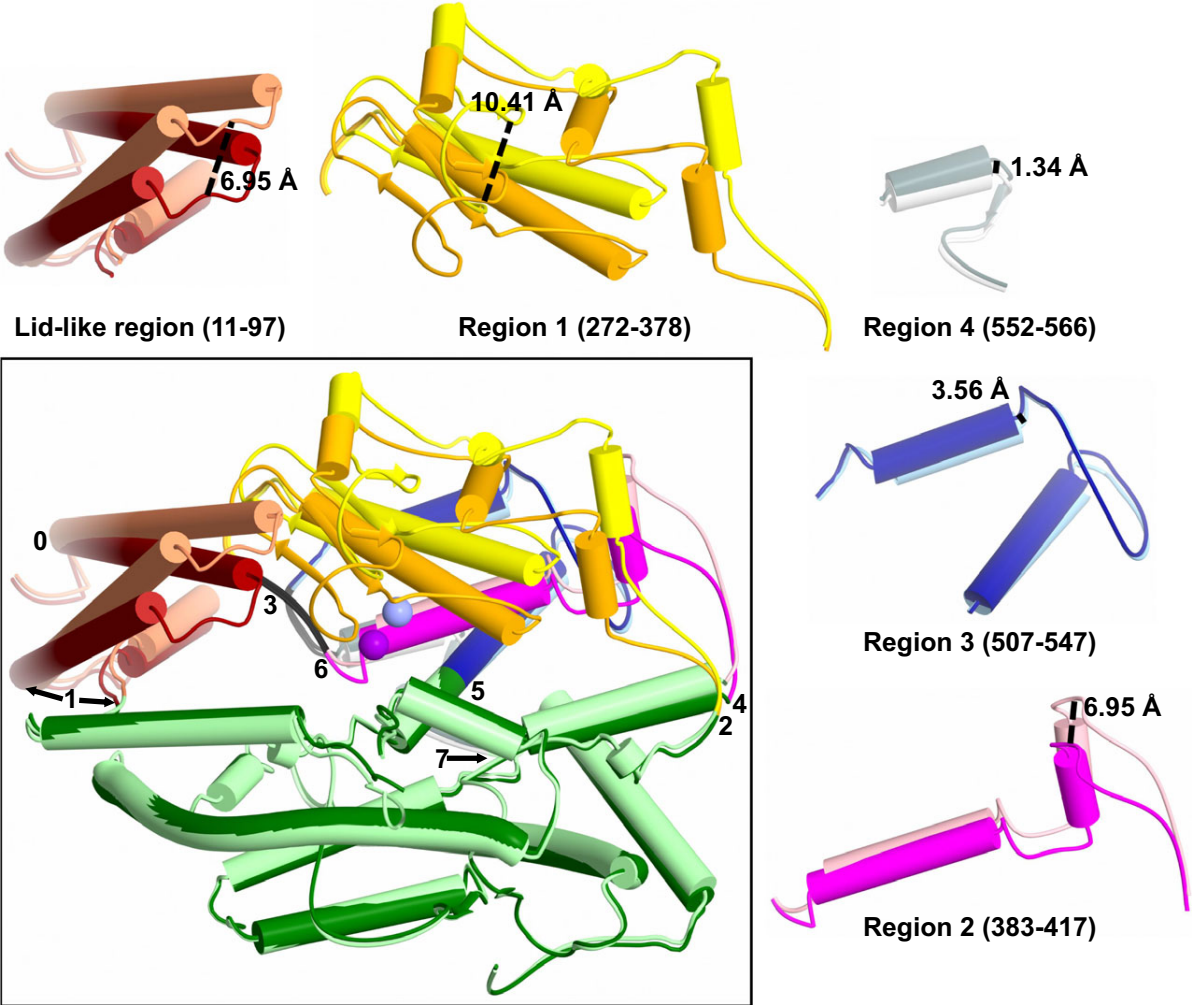
Mechanism of nACE opening. Overlay of the open and closed nACE structures highlighting the regions that move during the domain opening. Each moving region is also shown in isolation for clarity with the largest movement point marked. Subdomain 2 is shown in dark green/light green, with the moving regions of subdomain 1 coloured dark red/coral (residues 11–97), orange/yellow (residues 272–378), magenta/light pink (residues 384–417), dark blue/light blue (residues 507–547) and dark grey/light grey (residues 552–566). Zinc ion is shown as violet/lilac sphere. Darker colours are the closed structure. Figure was generated using CCP4mg [47].

Previous work using comparison of closed cACE structures with the open and closed ACE2 structures, followed by normal mode analysis identified six possible groups of residues (using nACE numbering, hinges 1 to 6 were residues 71–101, 274–275, 378–387, 412–417, 513–515 and 547–556, respectively) [29]. The new open nACE structure presented here allows for closer examination of overlays to provide more accurate identification of the hinge residues about which the subdomains pivot. While the open nACE structure largely agrees the hinge residues identified previously, detailed analysis revealed some modifications (Figs 8 and 9). The two subdomains are not formed by a simple N‐ and C‐terminal sections, but rather the peptide chain crosses between subdomain 1 and 2 several times. Likewise, while the overall effect of domain opening is to separate the two subdomains, the hinge regions are not confined to the residues on the subdomain junctions. When viewed from above the ‘clam shell‐like’ structure (with the subdomains being the two shell halves) and looking down into the binding site, the hinge regions are located at the bottom part of the structure, with the top edge of the subdomains moving the greatest distance (Figs 6A and 9).

To describe the movement involved in the domain opening in detail, subdomain 1 can be divided into sections that pivot away from subdomain 2 about hinge residues (Figs 8 and 9). The lid‐like region is largely comprised of two long helices (residues 14–44 H2 and 48–77 H3). When comparing the open and closed structures, the start of H2 (residue 14, labelled as hinge 0 to keep consistency with previously defined hinges 1–6) and end of H3 (residue 78) act as pivot points for these helices such that the H2‐to‐H3 loop moves by about 6.9 Å (colours dark red/coral, Fig. 9). The remaining part of the lid‐like region (residues 79 and 100) also moves, although only by a maximum of 2.2 Å, and can be considered to complete the hinge region (Hinge 1) for the pivot about residue 78. However, as observed in many closed ACE structures, and in particular the samASP‐nACE complex structure, there is a lot of flexibility in the lid‐like region. In contrast to the open/closed comparison, where the biggest movement is between H2 and H3, the general lid‐like flexibility shown in the samASP‐nACE complex was at the other end of the complex. This includes the first half of H2 (residues 9–28) and residues from the end portion of H3 until H5 (76–96) showing the maximum movement of about 3.5 Å and pivoting about residue 100. These regions are adjacent to a hole at the bottom of the nACE structure that leads into the nonprime binding lobe. While only one open structure is presented here, there is a similar, but small variation in the lid‐like region, particularly in residues 74–96, between the four molecules of the asymmetric unit. Interestingly, the hinge region around residue 100 (where the lid‐like region is connected to subdomain 2) is consistent for both subdomain opening and general lid‐like region flexibility indicating a crucial role. While the lid‐like region has been previously described as part of subdomain 1, it should perhaps be considered as a subdomain in its own right. This point is highlighted in Fig. 1A which shows that it is separate from the rest of subdomain 1.

The remainder of subdomain 1 can be split into 4 regions that each pivot about 2 hinge residues. The regions at the bottom of the structure only move a little, while the section at the top edge shows extensive rearrangement when compared to the closed structure (Fig. 9). At the bottom of subdomain 1, residues 552–566 (region 4—dark grey/light grey), which includes H26, only shift by up to 1.3 Å. While this degree of movement would not usually be significant when comparing structures, the shift in H26 directly matches the changes in helices above it in the structure and can therefore be considered as the base of the whole ‘clam shell‐like’ opening of the ACE domain. Therefore, while residues 547–556 were previously identified as hinge 6, it can now be more accurately described as residues 548–551, and a subtle but important new hinge is observed around residue 567 (hinge 7).

Hinge 6 is part of subdomain 1, rather than being on the interface with subdomain 2, and it is also the pivot point for region 3 (residues 507–547—dark blue/light blue), with hinge 5 (residue 507) being at the other end. This region sits directly above region 4 and has a larger movement of up to 3.6 Å. Region 2 (residues 384–417—magenta/light pink) pivots about hinges 3 (residues 378–384) and 4 (residue 417) giving a maximum movement of 6.6 Å, and it sits alongside region 3. It also contains Glu‐389, one of the zinc‐binding residues. Hinge 3 is part of subdomain 1, rather than at the interface with subdomain 2, and is instead adjacent to the lid‐like domain. Residue 378 of hinge 3 as well as hinge 2 (residue 272) provide the pivot points for region 1 (residues 272–378—orange/yellow), which sits above regions 2 and 3. This forms the top edge of subdomain 1 and therefore contains the largest degree of movement of up to 10.4 Å. Region 1 contains H16 with the zinc‐binding His‐361 and His‐365 residues, and as this is located towards the bottom of the groove, it results in a movement of 3.75 Å for the bound zinc ion.

As mentioned above, it is often possible to model multiple sugars of the glycosylation on Asn‐416 in closed nACE structures. This appears to result from a combination of crystal packing stabilising the positions of the Asn‐linked NAG and its 1–6 linked FUC, which orientates the sugar chain to pack against the protein surface residues 521–527 allowing hydrogen bond interactions with Tyr‐521, Glu‐522 and Gln‐527. Molecules in the asymmetric unit that lack the crystal packing stabilisation exhibit poorly defined electron density. In the open nACE structure, the crystal packing does not allow for any stabilisation of the Asn‐416 glycosylation. In addition, Asn‐416 is located on hinge 4 causing a change in orientation of the side chain, and residues 521–527 are located on the part of region 3 with the largest movement. These changes combine resulting in a highly flexible glycosylation group which, as stated above, is not possible to model.

### Comparison of nACE, cACE and ACE2 subdomain dynamics

The structure of ACE2, the second ACE homologue in humans, has previously been determined in both the closed and open conformations [26]. ACE2, nACE and cACE show 32% identity and 66% similarity between their sequences. When comparing ACE2 with the sACE domains individually, these values increase to 42% identity and 75% similarity with nACE, and 42% identity and 76% similarity with cACE. This high degree of sequence conservation means it is not surprising that the closed structure of the ACE2 domain has a similar overall secondary and tertiary structure (mainly α‐helical with two subdomains and a lid‐like region) as nACE and cACE (Fig. 6B). While an RMSD of 2.42 (570 Cα atoms) and 1.92 (578 Cα atoms) Å for nACE and cACE (Table 2), respectively, indicates some variation, this is largely present in the lid‐like region and some other loop regions.

The open nACE structure presented here allows for comparison with the open ACE2 structure to determine whether they adopt the same mechanism and subdomain dynamics to allow entry of substrates into the binding site. In the overlay of these two structures, we observed that the groove into the binding site of nACE is narrower than that of ACE2 (Fig. 6C), and this is highlighted by an RMSD of 2.88 Å for 571 Cα atoms. However, a detailed examination of the mechanism of opening shows they utilise the same subdomain dynamics (hinges and pivoting regions). Therefore, it can be concluded that the nACE open structure is slightly more closed than the ACE2 open structure. This raises the question of whether the nACE structure presented here is fully open or whether it crystallised in a partly open conformation.

A recent molecular dynamics study [28] using the Ang II‐cACE complex closed structure [34] showed that when the Ang II peptide was removed prior to a long 400‐ns simulation, the cACE structure went through various closed, partially open and fully open conformations. The most open cACE conformation observed was close to that of the open ACE2 structure. This suggests that the nACE structure presented here is in fact a partly open structure, which was either more favourable for crystallisation or was somehow prevented from opening further. Closer examination of the nACE open crystal structure revealed that the degree of opening may be less due to a magnesium ion‐mediated interaction between subdomain 1 Asp‐354 and subdomain 2 Glu‐431 (details of this interaction are described in the following Discussion section) as well as subdomain 1 His‐331 interacting with a bicine molecule that is part of the calcium ion/bicine complex bound in the subdomain 2 substrate C‐terminal binding site. Both of these interactions may combine to have the effect of linking the two subdomains and therefore help stabilise a partially open structure.

A previously reported BPPb‐cACE (bradykinin‐potentiating peptide b) complex structure showed that the zinc‐binding residues (His‐383, His‐387 and Glu‐411) underwent a conformational change and moved away from the usual ligand binding site, and it was concluded this may have been due to not having an active‐site zinc ion bound [34]. These residues are all located within subdomain 1 and can link the two subdomains when they bind a zinc ion through the bound ligand. We analysed this structure and observed that in fact there were conformational changes to the zinc‐binding residues, lid‐like region and other proximal loops which were similar, albeit smaller in magnitude, to those seen in the nACE and ACE2 open structures. We conclude that this cACE structure is also partially open and this is apparent when looking at overlay of these structures (Fig. 6D). Therefore, it can be concluded that nACE, cACE and ACE2 open using the same subdomain dynamics and that zinc ion is likely to be important in not only the catalysis itself, but also in forming the completely closed active conformation. By comparing a closed nACE structure with the BPPb‐cACE, open nACE and open ACE2 structures, we observed a gradual opening of the ACE domain (Fig. 7). It is likely that other ACE homologues, such as AnCE and AnoACE, are able to open in a similar way.

### Analysis of S′ subdomain interface‐interacting residues

Previous studies using the S_2__S′‐mutant nACE have shown that the nACE S′ Ser‐260, Glu‐262, Asp‐354, Ser‐357 and Glu‐431 residues (SEDSE which are mutated to cACE equivalents Thr, Ser, Glu, Val and Asp, respectively) distal from the active site are involved in interactions between the two subdomains and have suggested that their polar properties are involved in driving the closing of nACE [27, 30]. The native and S_2__S′‐mutant nACE open structures are presented here, along with the previously published closed structures of both, which allows closer examination of these residues at the subdomain interface for further insights into their role.

**Fig. 10:**
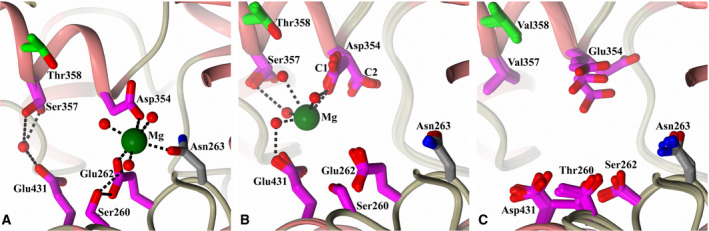
Comparison of the subdomain interface region. Close‐up view of the subdomain interface region of (A) typical closed nACE structure (PDB code‐6F9V [32]), (B) overlay of the four molecules in the open nACE crystal structure asymmetric unit (zinc ion and waters of only one molecule shown for clarity) and (C) overlay of the four molecules in the open S_2__S′‐nACE crystal structure asymmetric unit. S_2_′ and distal S′‐residues are coloured green and magenta, respectively. Figure was generated using CCP4mg [47].

In the closed conformation, all of the SEDSE residues can be involved in interactions between the two subdomains, although the different structures solved to date show that there is some flexibility resulting in variation of these interactions (Fig. 10A—example of maximum observed interactions shown in PDB 6F9V [32]), and this is likely affected by the conditions surrounding the enzyme. For example, both direct and water‐mediated interactions have been observed between Asp‐354 of subdomain 1 and Glu‐262 of subdomain 2. Alternatively, Asp‐354 and Glu‐262 can be involved in the coordination of a magnesium ion along with another subdomain 2 residue Asn‐263 (conserved in cACE) and three water molecules, one of which is also coordinated to subdomain 1 Ser‐260. In addition, Ser‐357 of subdomain 1 and Glu‐431 of subdomain 2 show a water‐mediated interaction. In contrast, in the S_2__S′‐mutant nACE only a single water‐mediated interaction is observed between Asn‐263 and Glu‐354, which is also present in cACE [30].

In the partially open conformation of native nACE, the greater distance between the two subdomains results in a significant reduction in interaction between the two subdomains through the SEDSE residues (Fig. 10B). Asp‐354 of subdomain 1 has two different conformations observed in the four molecules of the asymmetric unit, with either one or both of conformations observed in each individual molecule. One conformation of Asp‐354 (C2 Fig. 10B) results in lack of interactions between the two subdomains from the SEDSE residues. The closest distance of SEDSE residues between subdomains results from the other Asp‐354 conformation (C1 Fig. 10B) where its carboxylic acid side chain is 6.23 Å from the side chain of Glu‐431. Therefore, the only significant interaction possible at this distance is mediated by water or a magnesium ion, which is in a different location to that seen in the closed structures. It is interesting that the degree of opening observed for nACE occurs at the point where interaction between the subdomains through the SEDSE residues is at its limit. This potentially shows the importance of these residues that they may cause this partially opened conformation to be favoured over a more open conformation similar to ACE2, hence aiding crystallisation of this species.

Glu‐354 in the S_2__S′‐mutant nACE partially open structure shows multiple conformations in the four molecules of the asymmetric unit (Fig. 10C), indicating that it is more flexible and less constrained than the shorter Asp‐354 in the native structure. In one molecule, the distance from Glu‐354 to Asp‐431 is 5.86 Å and therefore closer than the 6.23 Å seen in the native open structure, while in the other conformations, the distance is greater. There are no water/ion‐mediated interactions observed between Glu‐354 and Asp‐431. While this may be partially due to the lower 2.80 Å resolution of the S_2__S′‐mutant nACE structure compared to the 1.85 Å of the native open structure, it is clear from the lack of electron density observed that even if the interactions were present, they would be weak.

The backbone of the closed structures of native and S_2__S′‐mutant nACE overlay well in general (RMSD 0.663 Å for 586 Cα atoms, Table 2) and in this region of the subdomain interface. Considering the reduced amount of localised subdomain interaction between the subdomains caused by the S′ mutations, it is possibly unexpected that the backbones of the open structures of native and S_2__S′‐mutant nACE also overlay closely (RMSD 0.425 Å for 586 Cα atoms, Table 2). However, the secondary structure of the individual subdomains is unlikely to be affected greatly by their surface residues (which include the S′ mutations when in an open conformation); therefore, it is the degree of opening that has to be considered. The S_2__S′‐mutant nACE crystals were grown by seeding from the native nACE crystals, and this could favour a similar partially open conformation due to crystal packing.

With the amount of domain opening seen in these crystal structures being at the limit of interaction of the SEDSE residues between the two subdomains, it seems likely that at this point, these residues in the native structure start contributing to the subdomain closure and this importance increases as the domain closes further. In contrast, the SEDSE residues in the S_2__S′‐mutant nACE require the subdomains to close further before they can give their reduced contribution at the subdomain interface that was concluded from the previous closed structures.

### ACE domain adaptations for substrate promiscuity

As described above, both sACE and ACE2 have been shown to hydrolyse a range of substrates or various lengths, with sACE also being able to catalyse both exo‐ and endopeptidase mechanisms. Analysis of previous structures and those presented here can be used to identify structural features that allow for these variations.

It has previously been suggested that the ‘lid‐like’ region of the ACE domains could control entry into the active site [24]. However, previous molecular dynamics studies and the open structures presented here show that the two subdomains open in a ‘clam shell‐like’ manner to allow binding of substrates and inhibitors. This indicates that the lid‐like region moves as part of the subdomains and that its involvement in the control of substrate entry/exit would just be a contribution at the edge of the nonprime side of the groove.

**Fig. 11:**
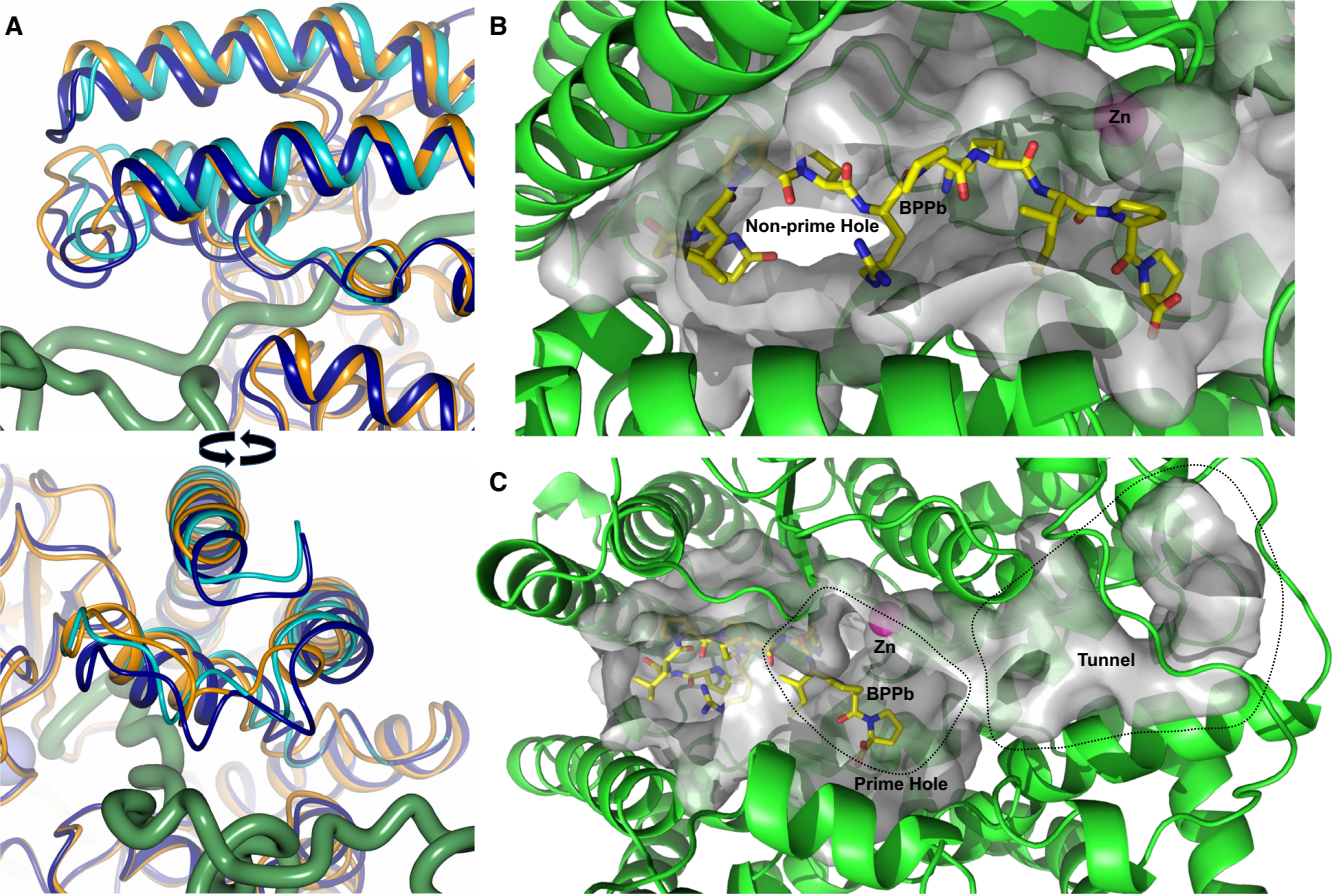
ACE structural adaptations for substrate promiscuity. (A) Flexibility of lid‐like region adjacent to C terminus insertion. Two views of the overlay of samASP‐nACE and C terminus insertion cACE structures showing how the flexibility observed in the lid‐like region has an effect on hole at the bottom of the nonprime lobe and could interact with peptides passing through this hole. The samASP‐nACE alternate lid‐like conformations are shown in light and dark blue, with cACE in orange. The C terminus of symmetry‐related cACE molecule is shown as green worm. (B and C) Accommodation of variation in peptide substrate size in ACE. Schematic representation of an overlay of nACE open structure with the BPPb peptide (yellow sticks) from an nACE complex structure (PDB 6QS1 [35]) showing (B) the hole at the base of the nonprime lobe, and (C) highlighting the possible routes for extension of the prime binding lobe. Binding groove/cavity is shown in grey with the active‐site zinc as magenta sphere. Panel A was generated using CCP4mg [47], with B and C using pymol [48].

The native cACE structure presented here proves that there is an alternative entry/exit point for peptides into the binding site region. The small hole and short tunnel observed here are of sufficient size for a peptide to pass through (Fig. 5). In addition, one end of the lid is adjacent to this entry point. This portion of the structure shows increased flexibility compared to most of the ACE structure; indeed, two conformations of this region were modelled in an nACE structure [32]. An overlay of the alternative nACE lid structure with the native cACE structure (Fig. 11A) shows about a 3 Å movement towards the inserted C terminus of the symmetry molecule. While this movement does not close off the entrance into the binding site, numerous residues (nACE 80–82 and 89–97, and cACE 104–106 and 89–97) are adjacent to the hole and have the potential to alter available space and provide interactions towards a peptide. These residues could therefore be involved in controlling the movement of peptides into and out of the binding site, indicating that this N‐terminal lid‐like region should be considered as separate from the rest of subdomain 1. Asn‐109 from the lid‐like region of cACE is glycosylated, and an extensive chain is observed in the native structure presented here where it is orientated such that it interacts with subdomain 2. The equivalent residue is not glycosylated in nACE due to it being Asp‐85, and it is part of the flexible region of the lid‐like structure observed in nACE. This suggests the possibility that this glycosylation could potentially stabilise and/or control the lid‐like region adjacent to the hole in cACE.

Most of the known substrates of ACE domains are relatively short, around 12 residues or less, and as shown by the 11‐mer BPPb bound to cACE and nACE, peptides of this length can fit entirely within the two‐lobed binding site [34, 35]. However, it has been unclear how longer peptide substrates, such as beta‐amyloids, could fit into the active site of ACE. While the open structure does show how peptide substrates could enter the active site, it does not fully explain how the long peptides could be accommodated, especially in the closed conformation. The native cACE structure presented here shows that peptides are able to extend through the hole at the end of the nonprime lobe opposite to the groove opening shown by nACE and ACE2 that allows peptide entry into the binding site. Long peptides could have their C terminus binding in the usual position in the prime lobe and their N terminus passing through the hole at the base of the groove, allowing the excess residues to be located outside the ACE protein.

An overlay of the BPPb peptide from a previously published nACE complex [35] with the open nACE structure shows that the BPPb N terminus is positioned approaching the hole in the nonprime lobe (Fig. 11B). This is consistent with longer peptides being able to adopt an orientation that allows them to pass through this hole described above. However, sACE is also known to be able to act as an endopeptidase for these longer peptides like Aβ [18, 21], but the closed structures of the sACE domains do not have much room available for further C‐terminal residues beyond the usual dipeptidase reaction. However, the overlay of the BPPb peptide with the open structure provides a potential explanation for this (Fig. 11C). Firstly, a view of the groove/cavity indicates that there is a potential tunnel from the peptide substrate C‐terminal binding site extending to the surface. However, this is quite narrow and would probably require the peptide to burrow through with some conformational changes. A potentially easier route would be the peptide substrate extending out the side of the groove on the prime side, where there is a large hole formed in the partially open cavity. This would require the ACE domain to wrap around the extended C terminus of the substrate adopting a conformational change in that region compared to typical closed structure. Further experimental study is required to fully understand how the structure adapts to allow for endopeptidase activity.

There are previously published structures where peptides have been hydrolysed, either during pre‐incubation with ACE or subsequent crystallisation, notably the Ang II and Aβ fragment complex structures [34, 36]. The products of these reactions have been shown bound and are either short dipeptides or tripeptides still bound in the prime lobe, or longer peptides such as Ang II that are nonprime products that have rebound after hydrolysis across both the prime and nonprime lobes. To date, no structures have been observed with cleaved products where the N‐terminal portion is bound solely in the nonprime lobe. We can therefore speculate that the nonprime products are able to exit the binding site, while the C‐terminal product remains bound. This could be because the C‐terminal products are more tightly held and so remain bound when the ACE domain opens and the N‐terminal product is released. Alternatively, the N‐terminal products may still be present in the nonprime lobe, but loosely bound because of their flexibility due to a lack of interactions and therefore not observed in the electron density maps. However, it is interesting to speculate that due to peptides being able to pass through the hole at the base of the nonprime lobe, the N‐terminal products may be able to exit this way. This could potentially mean that Ang II can be released more quickly by ACE and go on to affect the RAAS.

Finally, the previously published cACE structure in complex with the vasopeptidase inhibitor omapatrilat showed that in addition to the inhibitor binding in the usual S_1‐_S_2_′ subsites, an omapatrilat dimer bound in the nonprime lobe [37]. One half of this dimer occupies the same region of this lobe close to the hole as the C terminus insertion seen in this study. This supports the suggestion that inhibitors could possibly be designed solely targeting nonprime residues distal from the active‐site zinc ion, and due to the lower conservation between nACE and cACE in these regions, there is the potential for domain specificity. In addition, with the proximity of the hinge 1 region, there is the possibility of allosteric effects on both domain opening and the hole into the nonprime lobe through the important lid‐like region.

## Conclusion

We have crystallised the first open nACE structure and re‐analysed the previously published BPPb‐cACE structure, demonstrating that sACE opens with same subdomain dynamics as ACE2 to give a wide groove that allows peptides to easily access the binding site. Human sACE hydrolyses a wide range of peptide substrates of varying lengths with exo‐ and endopeptidase activity. The new structures presented here shed some light on how the ACE domains structurally achieve this diversity of activities. The cACE structure showing C terminus insertion from a symmetry‐related molecule explains how sACE is capable of cleaving long peptides that are too big to be enclosed within the nonprime binding lobe. This cACE structure also indicates that the hole in the nonprime lobe could be an additional exit route for N‐terminal products without the need for the enzyme to open. The open nACE structure suggests possible binding regions beyond the S_2_′ subsite that are required for the extended C‐terminal residues of a substrate undergoing endopeptidase cleavage. These regions distal from the active‐site core are potential targets for inhibitor design where they could be domain‐specific due to increased variation between nACE and cACE away from the active site, and also substrate‐specific due to the different peptide lengths. This targeted approach could lead to inhibitors with less side effects.

The shape of the groove in the open structure favours the peptide adopting an extended linear conformation, and there are likely to be interactions starting near the edge of the groove that is specific for the different peptide substrates that orientate and position them for the different types of hydrolysis. The binding of substrates and inhibitors to ACE domains is entropic [38], likely due to the displacement of water/solvent molecules by the ligand, and this would help drive the closure of the subdomains. In addition, analysis of the open native and S_2__S′‐mutant nACE structures highlights how residues distal to the S_2_′ subsite that are unique to nACE are able to effect affinity for some inhibitors by providing interactions between the two subdomains.

While these structures provide possible explanations for the endopeptidase activity and how a long peptide can be accommodated, it does not provide information on how this is achieved. Long peptides like beta‐amyloids will not readily adopt linear conformations in order to bind, so does the C terminus unravel as it passes through the hole in the nonprime lobe until it reaches and binds to the active‐site zinc ion and required C terminus binding subsite? Or are the residues at the lip of the open ‘clam shell‐like’ structure that interact with the peptide able to stabilise a linear conformation so that it can drop into the binding site? These questions should be further examined in future studies.

## Materials and methods

### Enzymes for crystallography

Minimally glycosylated N‐domain (N389) and C‐domain (g13) (Ser‐1 to Pro‐633) human ACE proteins and N389‐S_2__S′‐nACE were generated by expression in cultured mammalian CHO cells and purified to homogeneity as described previously [30, 31, 33].

### X‐ray crystallographic analysis

The ACE domains (8 mg·mL^−1^ G13‐cACE and 5 mg·mL^−1^ for both N389‐nACE and N389‐S_2__S′‐nACE, all in 50 mm Hepes, pH 7.5, 0.1 mm PMSF) were crystallised in hanging drops with 1 µL protein mixed with equal volume of well buffer (cACE 0.1 m MIB buffer, pH 4.0, 5% glycerol and 15% polyethylene glycol 3350, nACE 30% polyethylene glycol 550 MME/polyethylene glycol 20000, 0.1 m Tris/Bicine, pH 8.5, and 60 mm divalent cations, Molecular Dimensions Morpheus A9). Both proteins were then incubated at 16 °C where crystals of cACE readily grew. An atypical plate morphology crystal for N389‐nACE grew at the edge of a drop. After this was removed for X‐ray diffraction, further plate crystals grew in the same drop. These crystals were used to hair‐seed further N389‐nACE and N389‐S_2__S′‐nACE drops to produce the plate morphology crystals.

X‐ray diffraction data were collected on stations i03 (open nACE structure) and i04 (open S_2__S′‐nACE and cACE structures) at the Diamond Light Source (Didcot, UK). Crystals were kept at constant temperature (100 K) under the liquid nitrogen jet during data collection. Images were collected using a PILATUS3 6M and Eiger2 XE 16M detectors (Dectris, Baden‐Daettwil, Switzerland). Raw data images were indexed and integrated with DIALS [39] and then scaled using AIMLESS [40] from the CCP4 suite [41]. Initial phases for the native structure were obtained by molecular replacement with PHASER [42]. For the open nACE structure, individual subdomains (subdomain 1 residues 11–97, 272–417 and 507–566, and subdomain 2 residues 98–271, 418–506 and 567–601) of PDB code‐6F9V [32] were used as the search model, with the resulting refined structure being used for the open S_2__S′‐nACE structure. PDB code‐6F9U [32] was used as the search model for the cACE structure. Further refinement was initially carried out using REFMAC5 [43] followed by PHENIX [44], with Coot [45] used for rounds of manual model building. Zinc ions, purification/crystallisation buffer reagents and water molecules were added based on electron density in the *F*_o_‐*F*_c_ Fourier difference map. MolProbity [46] was used to help validate the structures. Crystallographic data statistics are summarised in Table 1. All figures showing the crystal structures were generated using either CCP4mg [47] or PyMOL [48], and schematic binding interactions are displayed using LigPlot^+^ [49].

### Accession numbers

The atomic coordinates and structure factors of the open native nACE, open S_2__S′‐nACE and native cACE from this work have been deposited in the Protein Data Bank under accession codes 6ZPQ, 6ZPT and 6ZPU, respectively.

## Conflict of interest

The authors declare no conflict of interest.

## Author contributions

GEC performed all the crystallographic analysis and wrote the manuscript. LL carried out the cloning, expression and purification of the S_2__S′‐mutant nACE and edited the manuscript. EDS edited the manuscript. KRA supervised the study, analysed the data and edited the manuscript. All authors reviewed the manuscript.
